# Navigating the pandemic in an acute-care hospital—The overlooked relationship between hospital leadership and infection prevention

**DOI:** 10.1017/ash.2022.249

**Published:** 2022-08-22

**Authors:** Jonas Marschall, Hilary M. Babcock, Urs P. Mosimann

**Affiliations:** 1 Department of Infectious Diseases, Bern University Hospital, Bern, Switzerland; 2 Division of Infectious Diseases, Washington University School of Medicine, St Louis, Missouri, United States; 3 BJC Healthcare, St Louis, Missouri, United States; 4 Medical Directorate, Bern University Hospital, Bern, Switzerland

Much has been written about the coronavirus disease 2019 (COVID-19) pandemic, both in scientific journals and in the lay press. Curiously, however, accounts or analyses of what contributed to the successful functioning of acute-care hospitals in these daunting times are rare.^1–4^ In addition, to our knowledge, there are no published assessments of what factors may have helped hospital leaders make optimal decisions for their institutions.

We believe that infection prevention (IP) programs have been well positioned to help acute-care hospitals navigate through the pandemic so far. Particularly when a mature IP program existed at the onset of the pandemic with good relationships with nursing and medical leaders at the facility, it likely increased the ability of the organization to keep up with and adapt to the high demand for critical decision making. To our surprise, we found only 2 electronic opinion articles that directly addressed this relationship.^5,6^

First, the chief medical officer (CMO), chief nursing officer (CNO), and other representatives of the hospital leadership needed easy access to basic epidemiological reasoning and had to learn and adopt these principles to make decisions for the hospital. We posit that this process was best managed in those institutions where there was an established, trusted relationship between medical leadership and IP, for whatever reason, even before the pandemic. Thus, the leadership could acquire needed knowledge quickly and from reliable partners and could develop some basic IP expertise themselves.^7^

Second, the CMO and CNO needed a group of experts around them that were independent and not overly guided by their own department’s particular interests. Infectious diseases divisions (and the IP programs that are usually led by infectious diseases physicians) most frequently operate clinically as consultants for all other specialties without having their own dedicated hospital beds. This makes them less vulnerable to negative financial impact during phases of reduced demand, as happened to some other specialties during the waves of the pandemic. Consequently, infectious diseases can adopt a high-level view of hospital operations more readily than other specialties and will not have to take the well-being of their clinical operations as the primary goal.

Third, IP experts are usually well connected with public health authorities, infection control bodies and other hospitals, and nearly all entities within their hospital such as emergency medicine and ICU teams, occupational health, patient safety, and even ventilation technicians. This connectivity and the resulting established human network positions them to be ideal counselors in critical situations because they can rely on pre-existing relationships with a variety of topical experts already working in the hospital.^8^ Notably, this network goes both ways: it can be used not only for gathering data but also for disseminating information or implementing measures.

Fourth, if there was a precedent of prior coordinated responses engaging leadership in infection prevention topics, then it was probably easier to get the COVID-19 response started and to find solutions to counter the waves of this pandemic. In our hospital group in Switzerland, for example, a large MDRO outbreak had led to the implementation of a dedicated task force a few years before the pandemic. It was chaired by the CMO, so there was already a common understanding of interdisciplinary crisis management that could easily be reactivated for the pandemic.^9^ Not only was the “old” task force revived, the CMO, CNO, and IP leadership also appeared jointly in town-hall–style meetings in front of hospital employees and projected a consistent, carefully crafted message. In essence, we believe that if a hospital’s readiness for handling outbreaks had already grown due to previous challenges that resulted in close collaboration between the hospital’s leadership and the IP program, then it is likely to have fared better during the COVID-19 pandemic response. For comparison, the level of preparedness of entire countries has been linked to previous outbreak experiences such as the 2015 MERS-CoV outbreak in South Korea and the 2003 SARS-CoV-1 outbreak in Taiwan.^10^

Fifth, the modus operandi of leadership throughout the pandemic has been one of developing several scenarios and opting for a “best guess” scenario to use as playbook. As Peter Kalina put it, “No previous training, no prior strategic planning knowledge, no prior operational experience, and no former decision-making skillset has prepared *anyone* for the uncertainty surrounding what this complex new reality presents us with.”^3^ In our opinion, the development of scenarios and the subsequent enforcement of measures that align with the anticipated course of the pandemic can best be achieved in the interplay between content experts (which includes the IP team) and decision makers (ie, the hospital leadership).

Finally yet importantly, the pandemic put a substantial stress on the workforce, including the professionals working in IP programs around the globe. Those programs that entered the pandemic era with an adequately staffed,^11^ well-educated, and experienced workforce, with a track record of working well together and with diverse expertise in various aspects of infection prevention and control, were probably better equipped to weather it. We also believe that those programs were less likely to lose team members to burnout. Again, the investment in personnel and resources for IP programs reflects the importance that is assigned to preventing infections by hospital leaders.

In conclusion, we believe the relationship between hospital leaders and IP leadership is critical to pandemic response preparedness and should be continuously cultivated in view of ongoing and likely future challenges due to infectious agents. A trustful relationship will encourage the CMO, the CNO and others to seek counsel with IP leaders. The holistic perspective on a complex system is a characteristic of both hospital and IP leadership and should be employed to the benefit of a given healthcare institution and its care processes.^12^ IP experts who work closely with clinical caregivers and many other services throughout the hospital can help the hospital leadership maintain a 360° view and can help implement important measures to make operations as resilient as possible.^13^
